# Alpinetin inhibits neuroinflammation and neuronal apoptosis via targeting the JAK2/STAT3 signaling pathway in spinal cord injury

**DOI:** 10.1111/cns.14085

**Published:** 2023-01-10

**Authors:** Shining Xiao, Yu Zhang, Zihao Liu, Anan Li, Weilai Tong, Xu Xiong, Jiangbo Nie, Nanshan Zhong, Guoqing Zhu, Jiaming Liu, Zhili Liu

**Affiliations:** ^1^ Department of Orthopedics The First Affiliated Hospital of Nanchang University Nanchang China; ^2^ Institute of Spine and Spinal Cord Nanchang University Nanchang China; ^3^ Medical Innovation Center The First Affiliated Hospital of Nanchang University Nanchang China; ^4^ Department of General Surgery The First Affiliated Hospital of Nanchang University Nanchang China

**Keywords:** Alpinetin, JAK2/STAT3 pathway, neurinflammation, neuronal apoptosis, spinal cord injury

## Abstract

**Background:**

A growing body of research shows that drug monomers from traditional Chinese herbal medicines have antineuroinflammatory and neuroprotective effects that can significantly improve the recovery of motor function after spinal cord injury (SCI). Here, we explore the role and molecular mechanisms of Alpinetin on activating microglia‐mediated neuroinflammation and neuronal apoptosis after SCI.

**Methods:**

Stimulation of microglia with lipopolysaccharide (LPS) to simulate neuroinflammation models in vitro, the effect of Alpinetin on the release of pro‐inflammatory mediators in LPS‐induced microglia and its mechanism were detected. In addition, a co‐culture system of microglia and neuronal cells was constructed to assess the effect of Alpinetin on activating microglia‐mediated neuronal apoptosis. Finally, rat spinal cord injury models were used to study the effects on inflammation, neuronal apoptosis, axonal regeneration, and motor function recovery in Alpinetin.

**Results:**

Alpinetin inhibits microglia‐mediated neuroinflammation and activity of the JAK2/STAT3 pathway. Alpinetin can also reverse activated microglia‐mediated reactive oxygen species (ROS) production and decrease of mitochondrial membrane potential (MMP) in PC12 neuronal cells. In addition, in vivo Alpinetin significantly inhibits the inflammatory response and neuronal apoptosis, improves axonal regeneration, and recovery of motor function.

**Conclusion:**

Alpinetin can be used to treat neurodegenerative diseases and is a novel drug candidate for the treatment of microglia‐mediated neuroinflammation.

## Graphical abstract

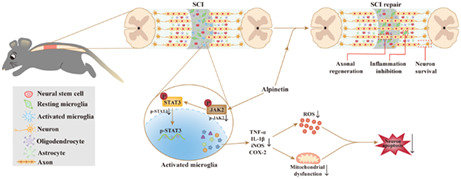

## 1 INTRODUCTION

Spinal cord injury (SCI) is a serious neurological disorder that can result in the loss of sensory and motor function.^1^, ^2^ Traumatic events, such as motor vehicle accidents, can lead to the compression and tearing of spinal cord tissue, referred to as primary injury.^2^ This is followed by secondary injury, which consists of inflammation, apoptosis, oxidative stress, neuronal loss, and axonal injury,^3^, ^4^ ultimately leading to massive tissue loss and motor dysfunction. The resulting inflammatory response can amplify secondary damage to the spinal cord and affect tissue repair.^5^, ^6^ The primary inflammatory mediators in the central nervous system (CNS) are microglia.^7^

Under physiological conditions, microglia‐mediated neuroinflammation is fundamental for the protection of the CNS, helping to maintain its stability.^8^ However, microglia are prone to overactivation when exposed to abnormal stimuli, such as SCI^9^, ^10^ and neurotoxins.^11^, ^12^ This can result in the release of numerous inflammatory mediators, including tumor necrosis factor‐alpha (TNF‐α), cyclooxygenase‐2 (COX‐2), interleukin‐1 beta (IL‐1β), and inducible nitric oxide synthase (iNOS).^8^, ^13^, ^14^ These pro‐inflammatory substances increase the aberrant generation of ROS^15^, ^16^ and cause the alterations in MMP in neurons,^17^, ^18^ leading to neuronal damage and death. Such effects can accelerate and worsen the progression of CNS diseases such as SCI.^19^, ^20^ Accordingly, inhibiting microglial activation and the subsequent inflammatory response in damaged areas represents a key treatment strategy for the recovery of patients with SCI.

In animal models of SCI, antineuroinflammatory drugs show some effect in improving motor function.^21^, ^22^ Over recent years, monomers derived from Chinese herbal medicines have received widespread attention in the treatment of SCI because of their low toxicity and reduced side effects.^23^, ^24^ Alpinetin is a natural flavonoid and the main active ingredient of *Alpinia katsumadai* Hayata, a traditional medicinal plant.^25^ This flavonoid has been used in the treatment of a variety of conditions, including inflammation,^26^ cancer,^27^ liver disease,^28^ and brain disease.^29^ In multiple inflammatory models, Alpinetin modulates the ERK/JNK/p38 MAPK, TLR4/NF‐κB, Nrf2/HO‐1, and PI3K/AKT signaling pathways to produce strong antiinflammatory effects.^30^ However, it is unknown if Alpinetin can control microglial activation and have neuroprotective benefits in SCI.

In this research, we discovered that Alpinetin exerts antineuroinflammatory effects by inactivating the Janus kinase (JAK)/signal transducer and activator of transcription (STAT) signaling pathway via targeting JAK2. Alpinetin treatment also alleviated neuroinflammation‐mediated abnormal ROS production and MMP changes in neuronal cells, while achieving a neuroprotective effect. In vivo, we demonstrated that Alpinetin can inhibit the inflammatory response and neuronal apoptosis in the damaged area after SCI, thereby promoting axonal regeneration and ultimately improving motor function recovery. These data suggested that Alpinetin may be a potential neuroprotective agent with antineuroinflammatory effects that could be used in the treatment of neurodegenerative diseases.

## 2 METHODS

### 2.1 Reagents and antibodies

Alpinetin (CAS. 36052‐37‐6, purity ≥98%) was purchased from Chemstrong Scientific Co., Ltd. Cy3‐labeled goat anti‐rat IgG, Alexa Fluor 555‐tagged donkey antirabbit/mouse IgG, Alexa Fluor 488‐tagged goat antirabbit/mouse IgG, horseradish peroxidase‐labeled secondary antibodies, antifluorescence quenching agent (containing DAPI), and CCK‐8 reagent were purchased from Beyotime. Lipopolysaccharide (LPS) was purchased from Merck. Anti‐COX‐2, anti‐Iba‐1, anti‐CD68, anti‐GFAP, and anti‐GAPDH antibodies were purchased from Abcam. Primary antibodies against iNOS, p‐JAK2, p‐STAT3, STAT3, β‐actin, Bcl‐xL, GFAP, NeuN, GAP43, and MAP2 were obtained from Cell Signaling Technology. Alexa Fluor 488 phalloidin was also from CST. The anti‐CD11b antibody was bought from Biolegend. The anti‐JAK2 antibody was provided by Affinity. WP1066 purchased from GlpBio. Transwell chamber was purchased from Corning (0.4 μm pores). Calcein AM/propidium iodide (PI) was bought from Solarbio. The JC‐1 MMP assay kit and the ROS fluorescence test kit were from Elabscience. The polymerase chain reaction (PCR) primers were synthesized by Servicebio while the kit for RNA extraction came from EZBioscience. The reagents for quantitative real‐time PCR (qPCR) were purchased from Vazyme.

### 2.2 Rat model of SCI and drug treatment

For animal experiments, 54 adult female Sprague Dawley rats (~200 g) were obtained from Hunan Silaikejingda Experimental Animal Co., Ltd. For modeling, rats were anesthetized with 1% (*w*/*v*) sodium pentobarbital (40 mg/kg). The rats were randomly divided into three groups, namely, a SCI (model) group, an Alpinetin group, and a Sham group. In the model group, the spinal cord was exposed with T9 as the center and a crush injury was generated in spinal cord tissue by clamping with a vascular clip for 10 s. In the Alpinetin group, Alpinetin (30 mg/kg) was intraperitoneally injected immediately after SCI. In the Sham group, the spinal cord was exposed, but no injury was made. After surgery, the bladder of the injured rats was emptied twice a day. After surgery, rats in the Alpinetin group were intraperitoneally injected with Alpinetin (30 mg/kg) into the spinal cord for 3 consecutive days.

### 2.3 Behavioral assessment

Motor function after SCI was assessed according to the Basso Beattie Bresnahan (BBB) locomotion rating scale, footprint analysis, and electrophysiology. BBB scores varied from 0 (total paralysis) to 21 (normal exercise). BBB scores were performed at 1, 3, 7, 14, 21, 28, 35, 42, 49, and 56 days after SCI to assess the extent of recovery of the hind limbs of the rats after injury. Footprint testing was performed 28 and 56 days after surgery for each group of rats. In addition, motor‐evoked potentials (MEPs) of rats were measured on Day 56 post‐surgery. Briefly, after anesthesia, two stimulation electrodes were placed in the cerebral cortex motor area and the recording electrode was inserted into the anterior tibial muscle of the contralateral hindlimb to record the hindlimb response in the rats. Changes in the amplitude of the MEP were recorded.

### 2.4 Histopathology

Heart, liver, spleen, lungs, kidneys, and spinal cord tissue were preserved in 4% PFA, paraffin‐embedded, and cut into 4‐5‐μm‐thick longitudinal and transverse sections. After that, the sections underwent Nissl and hematoxylin and eosin (H&E) staining. Images were acquired using a pathology slide scanner (Olympus).

### 2.5 Cell culture

Procell provided BV2 and PC12 cell lines. The cells were cultured with high‐glucose DMEM supplemented with 10% fetal bovine serum (Gibco, Thermo), 100 U/ml penicillin, and 100 μg/ml streptomycin. Cells were kept alive at 37°C in 5% CO_2_ incubator. Cells were passaged and treated at 80% confluence. The medium was changed every 2 days, and the cell growth was observed under the inverted microscope.

### 2.6 Co‐culture of microglia and neurons

To assess the impact of Alpinetin on microglia‐induced neuronal death in vitro, BV2 microglia and neuronal PC12 cells were co‐cultured using the Transwell system (0.4 μm pore, 6‐well plates). Before co‐culture, BV2 cells were pretreated overnight with Alpinetin (0, 50, or 100 μg/ml) and then stimulated or not with 1 μg/ml LPS for 24 h, resulting in varying degrees of microglial activation. To avoid any effects of LPS and Alpinetin on PC12 cells, the BV2 cells were first rinsed with PBS, and then co‐cultured with PC12 cells. After 24 h of co‐culture (2 × 10^5^ BV2 cells in the upper chamber of the Transwell inserts and 5 × 10^5^ PC12 cells in the bottom chamber of the inserts), the protective effects of Alpinetin on neurons were assessed using immunofluorescence and Western blot.

### 2.7 CCK‐8 assay

Microglia were treated with different doses of Alpinetin (0, 12.5, 25, 50, 100, 200, or 400 μg/ml) for 24 h after seeding in 96‐well plates, and then add 10% CCK‐8 reagent to incubate for 2 h. Finally, a microplate reader (Thermo) was used to detect the optical density at a wavelength of 450 nm.

### 2.8 MMP detection

A JC‐1 assay kit was used to detect MMP. JC‐1 acts as a fluorescent probe that can quickly detect changes in the MMP, thereby serving as an early marker of cell apoptosis. PC12 cells in the co‐culture system were incubated with JC‐1 reagent for 30 min in the dark. Using an inverted fluorescence microscope (ZEISS), take images of PC12 cells after removing the dye solution.

### 2.9 Intracellular ROS detection

Dichloro‐dihydro‐fluorescein diacetate (DCFH‐DA, 10 μM) was treated with PC12 cells for 30 min in the dark at 37°C. After rinsing three times with buffer solution, the intracellular ROS concentration was checked using an inverted fluorescence microscope (ZEISS).

### 2.10 Live/dead staining

The PC12 cells in the co‐culture system were treated with 1 μM calcein AM and 2.5 μM PI for 15 min at 37°C. Using an inverted fluorescence microscope (ZEISS), take images of live/dead PC12 cells after removing the dye solution.

### 2.11 qPCR

To evaluate the mRNA expression levels of IL‐1β, TNF‐α, COX‐2, and iNOS, RNA was isolated from BV2 cells using an RNA extraction kit, reversed transcribed, and the resulting cDNA was subjected to qPCR. GAPDH was used for normalization. Table 1 contains a list of the primer sequences utilized in this study.

**TABLE 1:** Primers used for quantitative real‐time PCR analysis.

Gene	Forward	Reverse
iNOS	CAACAGGAACCTACCAGCTCACT	AGCCTGAAGTCATGTTTGCCG
COX‐2	GAAATATCAGGTCATTGGTGGAGA	ATGCTCCTGCTTGAGTATGTCG
TNF‐α	AGACCCTCACACTCACAAACCA	CTTTGAGATCCATGCCGTTG
IL‐1β	AGGCTCCGAGATGAACAACAAA	GTGCCGTCTTTCATTACACAGGA
GAPDH	CCTCGTCCCGTAGACAAAATG	TGAGGTCAATGAAGGGGTCGT

### 2.12 Western blot

The RIPA lysis buffer was used to separate proteins from tissues and cells. Then SDS‐PAGE was used to separate the protein sample and then transferred to PVDF membrane and 5% skimmed milk for sealing. Then, incubate the membrane overnight at 4°C with primary antibody against iNOS (1:1000), COX‐2 (1:2000), JAK2 (1:2000), phosphorylated (p)‐JAK2 (1:1000), STAT3 (1:1000), p‐STAT3 (1:1000), Iba‐1 (1:2000), CD68 (1:2000), NeuN (1:1000), Bcl‐xL (1:1000), GAP43 (1:1000), MAP2 (1:1000), GAPDH (1:4000), and β‐actin (1:1000). On the second day, the membrane was incubated with horseradish peroxidase bound secondary antibody at room temperature for 1 hour. After washing, membranes were detected applying a chemiluminescent reagent and visualized using Image Lab software (Bio‐Rad). ImageJ was employed for quantitative analysis.

### 2.13 Immunofluorescence staining

In vitro, BV2 and PC12 cells were seeded at a density of 3 × 10^5^ cells/ml onto round glasses (20‐mm diameter) previously placed in a 6‐well plate, treated, and subjected to immunofluorescence staining. Briefly, the primary antibodies against Iba‐1 (1:300), CD11b (1:100), p‐STST3 (1:300), Bcl‐xL (1:200), and phalloidin (1:500) were incubated overnight at 4°C after the cells had been fixed in 4% paraformaldehyde (PFA), permeabilized with 0.5% Triton X‐100, blocked with 5% bovine serum albumin.

The next day, the cells were incubated with the respective fluorescent secondary antibodies for 2 h at room temperature. After washing, the coverslips containing the cells were placed on slides, an antifluorescence quenching agent (including DAPI) was added, and the coverslips were sealed. Images were captured by confocal laser scanning microscopy (Leica, Germany) and analyzed using ImageJ software.

For tissue sections in vivo, they were dewaxed in xylene, rehydrated in a graded ethanol series, blocked with 5% bovine serum albumin for 2 h at room temperature, and then incubated with primary antibodies overnight at 4°C. For longitudinal sections, the following antibodies were used: anti‐Iba‐1 (1:300), anti‐CD68 (1:500), anti‐GFAP (1:500), anti‐NeuN (1:200), anti‐Bcl‐xL (1:200), anti‐MAP‐2 (1:200), and anti‐GAP43 (1:300). For transverse sections, the antibodies targeted Iba‐1 (1:300), CD68 (1:500; Abcam), and GFAP (1:500; Abcam). The next day, the sections were incubated with Alexa Fluor 555‐ and Alexa Fluor 488‐conjugated secondary antibodies at 37°C for 1 h. Before sealing the slide with a coverslip, add the fluorescence quenching agent (containing DAPI), and then use a confocal laser scanning microscope (Leica) to take an image.

### 2.14 Network pharmacology and bioinformatic analysis of Alpinetin

The SwissTargetPrediction (http://swisstargetprediction.ch/), SEA databases (http://sea.bkslab.org/), and HERB databases (http://herb.ac.cn/) were used to predict the target proteins of the Alpinetin monomer (PubChem CID: 154279). Additionally, inflammatory disease‐related proteins were identified via the GeneCards, NCBI, and OMIM databases. Target proteins present in both groups were considered to be potential targets of Alpinetin in inflammation. The overlapping target proteins were also uploaded to the KEGG database for the building of a network of signaling pathways. To analyze drug target interactions associated with Alpinetin, the overlapping targets were also imported into the STRING database and Cytoscape for protein–protein interaction (PPI) network construction. Next, key proteins were screened by topological analysis and MCODE clustering analysis. AutoDock Vina software was used for the docking of Alpinetin with the JAK2 protein (protein number: 4IVA) and for calculating the binding free energy.

### 2.15 Statistical analysis

All statistical analyses were performed using GraphPad Prism 8.0. The means ± standard deviation of the data is displayed. Shapiro–Wilk was used to test whether the data distribution was normal. For normal distribution data, Tukey's multiple‐comparison test was used in conjunction with one‐way analysis of variance (ANOVA). Statistics are deemed significant when *p < 0.05. *p < 0.05, **p < 0.01 or ***p < 0.001.

## 3 RESULTS

### 3.1 Alpinetin inhibited the release of pro‐inflammatory cytokines in LPS‐activated microglia

**FIGURE 1:**
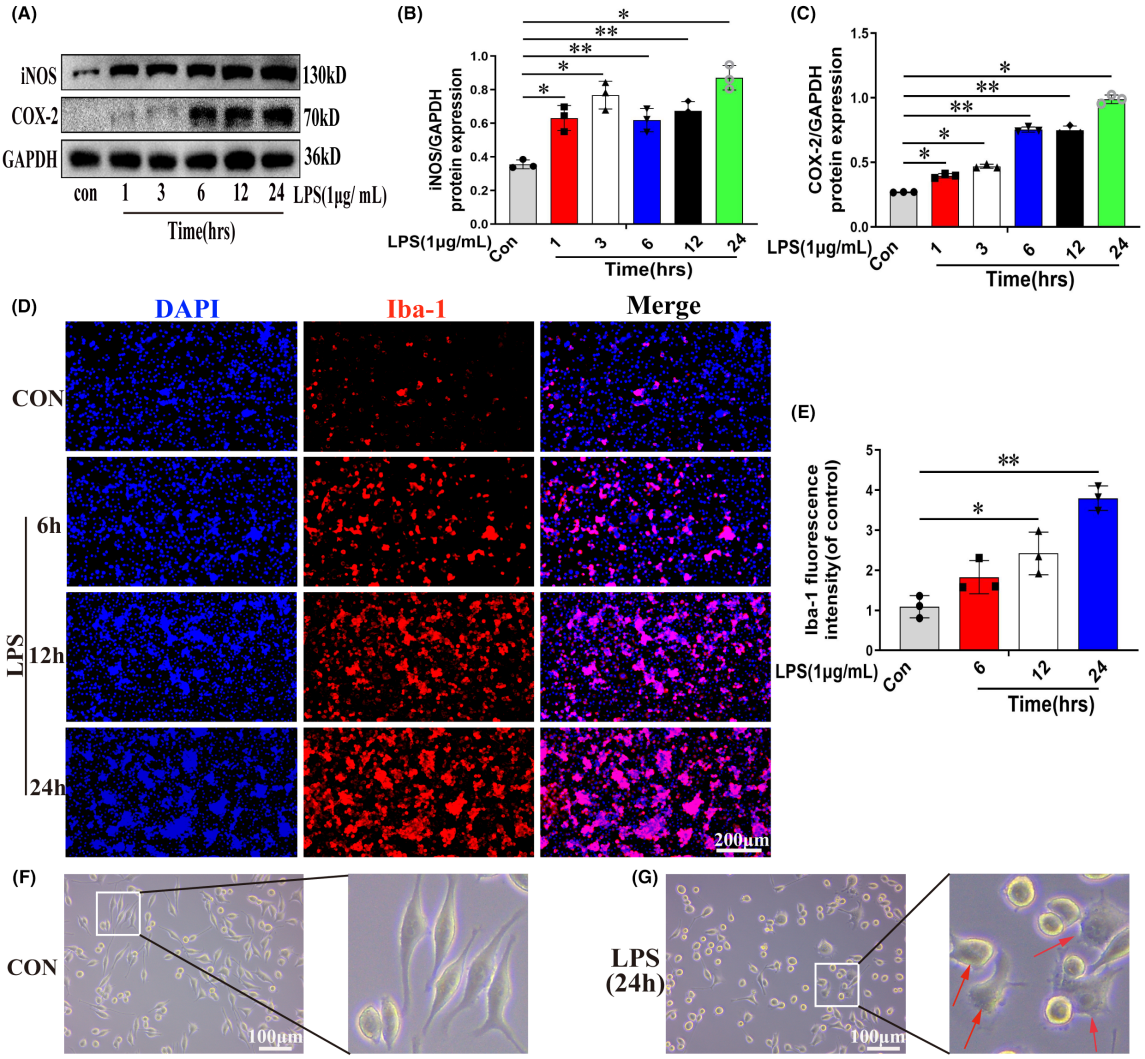
Lipopolysaccharide (LPS) triggers microglial activation in a time‐dependent manner. (A–C) Western blot images and quantification showing iNOS and COX‐2 protein levels in microglia. (D, E) Immunolabeling and quantification of Iba‐1 intensity in microglia. (F, G) Changes in the morphology of microglia with or without LPS stimulation. *N* = 3 per group for Western blot assay, *N* = 3 per group for Immunofluorescence staining assay. *p < 0.05 and **p < 0.01.

LPS (1 μg/ml) can reportedly activate BV2 cells and can thus be used to construct a model of neuroinflammation in vitro.^8^, ^31^, ^32^ First, we determined the optimal time point for the induction of neuroinflammation by LPS. As shown in Figure 1A–C, compared with the control (CON) condition, the protein expression levels of iNOS and COX‐2 increased in a time‐dependent manner from 1 h after LPS stimulation and peaked after 24 h (*p* < 0.05). To further confirm these findings, we performed immunostaining for Iba‐1, a microglial surface marker that is upregulated during SCI‐induced microglial activation.^3^, ^33^ The Iba‐1 immunostaining results were consistent with those of Western blotting (Figure 1D,E; *p* < 0.05). We further examined the morphology of LPS‐treated microglia after 24 h of exposure and found that the cells displayed an amoeba‐like morphology (shown by the red arrow) (Figure 1F,G). These results suggested that LPS stimulation can greatly induce the activation of BV2 microglia and promote the release of inflammatory mediators by these cells and that the levels of the mediators peaked after 24 h.

**FIGURE 2:**
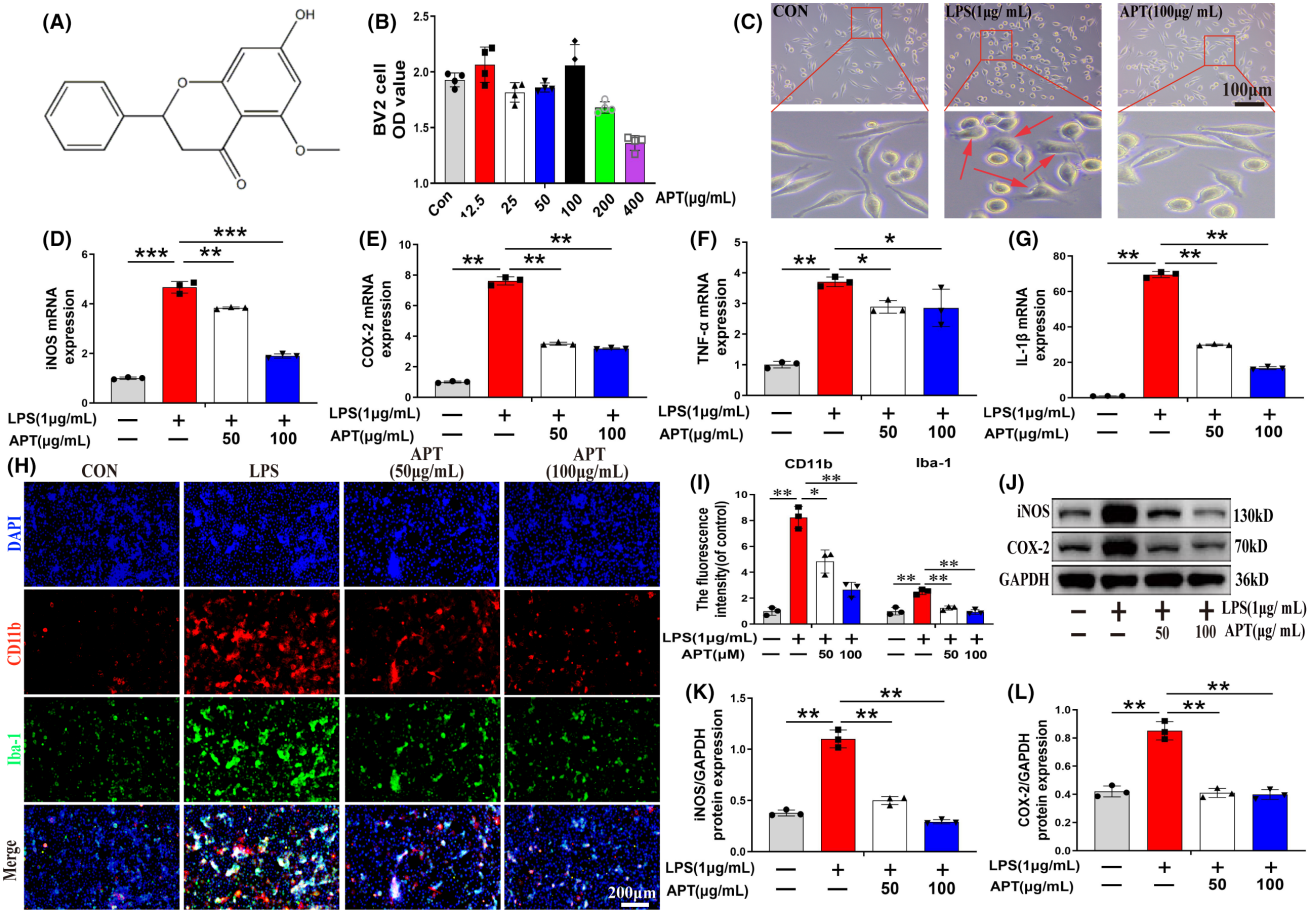
Alpinetin inhibits the release of proinflammatory mediators in lipopolysaccharide‐activated microglia. (A) The chemical structure of Alpinetin. (B) CCK8 assay of Alpinetin on BV2 viability for 24 h (control group is treated without Alpinetin). (C) Morphological changes in microglia. (D–G) The expressions of iNOS, COX‐2, TNF‐α and IL‐1β were assessed by RT‐qPCR. (H, I) Immunofluorescence staining and quantitative data revealed the expression levels of CD11b and Iba‐1 in microglia. (G–L) Western blot images and quantification showing iNOS and COX‐2 protein levels in microglia. *N* = 3 per group for Western blot assay, *N* = 3 per group for Immunofluorescence staining assay. *p < 0.05, **p < 0.01 and ***p < 0.001.

Next, we assessed the level of Alpinetin toxicity toward BV2 cells. Alpinetin's chemical structure is shown in Figure 2A. Alpinetin was applied to BV2 cells in a range of concentrations (0, 12.5, 50, 100, 200, and 400 μg/ml) for 24 h, and the CCK8 test was used to measure the effect on microglial viability. The results showed that Alpinetin concentrations below 100 μg/ml were not significantly toxic to microglia, whereas BV2 cell viability was significantly decreased at Alpinetin concentrations of 200 and 400 μg/ml (Figure 2B). Accordingly, in this study, Alpinetin concentrations of 100 μg/ml or less were used for subsequent experiments involving BV2 cells. Microscopic observation further indicated that LPS stimulation led to morphological changes in microglia, manifested as amoeba‐like changes (indicated by the red arrow); however, these changes were not observed when the cells were pretreated with Alpinetin (Figure 2C). These findings suggested that Alpinetin can significantly inhibit microglial activation.

In order to determine the impact of Alpinetin on inflammatory mediators in LPS‐activated microglia, we first used qPCR to assess the expression levels of genes encoding pro‐inflammatory mediators. As shown in Figure 2D–G, compared with controls, LPS treatment (1 μg/ml) significantly increased the mRNA levels of iNOS, COX‐2, TNF‐α, and IL‐1β, whereas pretreatment with Alpinetin blocked these increases in a concentration‐dependent manner (*p* < 0.05). To further confirm these findings, we performed dual immunostaining for CD11b and Iba‐1 (both microglial surface markers) to assess the degree of microglial activation. We found that exposure to LPS upregulated the expression of Iba‐1 and CD11b in microglia, indicating that they had been activated. However, Alpinetin pretreatment markedly suppressed these effects (Figure 2H,I; *p* < 0.05). Subsequently, Western blot analysis showed that pretreatment with Alpinetin (50 and 100 μg/ml) blocked the LPS‐induced increase in the protein expression of iNOS and COX‐2 in a concentration‐dependent manner (Figure 2J–L; *p* < 0.01). Overall, these results suggested that APL exerted significant antineuroinflammatory effects by inhibiting the overactivation of microglia and generation of inflammatory mediators.

### 3.2 Alpinetin reduced the levels of pro‐inflammatory cytokines in LPS‐induced microglia by inactivating the JAK/STAT pathway

To further explore the molecular mechanisms by which APL inhibits neuroinflammation in microglia, we obtained 142 predicted protein targets of APL through the SwissTargetPrediction, SEA, and HERB databases. Meanwhile, a total of 100 common targets were identified from among the 3678 genes associated with inflammatory diseases obtained through the GeneCards, NCBI, and OMIM databases (Figure S1A). Next, KEGG pathway enrichment analysis was performed on the overlapping targets (predicted Alpinetin targets and inflammatory disease‐related proteins), leading to the identification of 159 signaling pathways. These were then sorted by the number of target proteins (counts) in the pathways to screen the top 40 pathways (Figure S1B). Based on the literature, we found that the HIF‐1,^34^ TNF,^35^ PI3K/AKT,^36^ IL‐17,^37^ VEGF,^37^ and JAK/STAT signaling pathways^38^, ^39^ were involved in the regulation of microglia‐mediated inflammatory responses. Accordingly, we initially selected these six signaling pathways for further study. In addition, we built a PPI network based on the common targets (Figure S1C,D) and then screened the core targets through topological and MCODE clustering analysis. We identified 20 and 3 core targets, respectively, and, after excluding duplicate proteins, a total of 22 core targets were obtained (Figure S1E,F). The JAK/STAT pathway was chosen for follow‐up experiments because it was the most enriched, at 70%.

**FIGURE 3:**
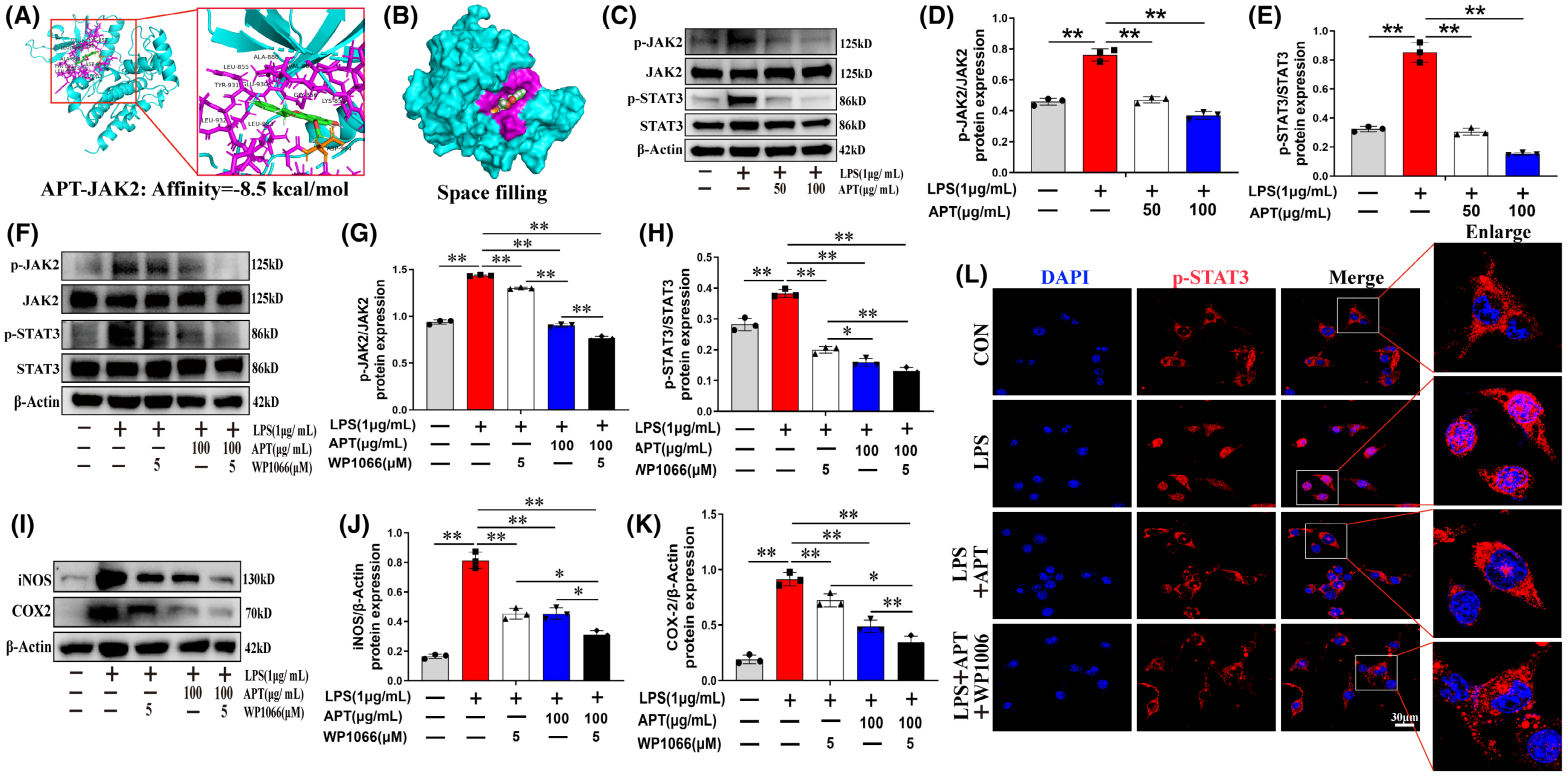
Alpinetin inhibit lipopolysaccharide‐mediated neuroinflammatory response in microglia via targeting JAK2/STAT3 signaling pathway. (A) Molecular docking of Alpinetin molecule and JAK2 protein was performed using Autodock vina. (B) Space filling model showing Alpinetin binding in the JAK2 binding pocket. (C–H) Western blot images and quantification showing p‐JAK2, JAK2, p‐STAT3, and STAT3 in microglia. (I–K) Western blot protein expressions and quantification data of iNOS and COX‐2. (L) The intracellular localization of p‐STAT3 (red fluorescence) was detected by immunofluorescence. *N* = 3 per group for Western blot assay, *N* = 3 per group for Immunofluorescence staining assay. *p < 0.05 and **p < 0.01.

Next, we explored whether Alpinetin modulates LPS‐induced neuroinflammation through the JAK/STAT pathway. JAK2 and STAT3 are key proteins in this pathway,^40^ playing an important role in multiple CNS pathologies, such as Alzheimer's disease,^41^ Parkinson's disease,^42^ and cerebral ischemic disorders.^43^ Additionally, JAK2 was identified as a common core target by topological and MCODE clustering analysis. We applied AutoDock Vina to molecularly dock Alpinetin (PubChem CID: 154279) and JAK2 protein (protein number: 4IVA) and found that Alpinetin could bind to the active pocket of JAK2 (Figure 3A,B) and that this binding was stable (binding energy: −8.5 kcal/mol). JAK2 and STAT3 phosphorylation levels in BV2 microglia were considerably increased by LPS treatment according to Western blot study, effects that were blocked by Alpinetin pretreatment (Figure 3C–E; *p* < 0.01). To further evaluate the function of JAK2/STAT3 signaling in the Alpinetin‐mediated reduction of pro‐inflammatory mediator release, we pretreated microglia with Alpinetin (0 or 100 μg/ml) and, before applying LPS, the cells were incubated or not with the JAK2/STAT3 pathway inhibitor WP1066 (5 μM) for 2 h. As shown in Figure 3F–H, combination therapy with WP1066 and Alpinetin resulted in a significant reduction in the phosphorylation of JAK2 versus STAT3 compared with that seen with WP1066 or Alpinetin monotherapy (*p* < 0.05). Similarly, the combined application of WP1066 and Alpinetin significantly downregulated iNOS and COX‐2 protein levels compared with WP1066 or Alpinetin treatment alone (Figure 3I–K; *p* < 0.05). We further evaluated the effect of Alpinetin on p‐STAT3 localization in activated microglia via immunofluorescence staining. After LPS treatment, STAT3 phosphorylation was enhanced, and an increase in p‐STAT3 nuclear translocation was observed (Figure 3L). However, Alpinetin pretreatment significantly blocked the LPS‐induced increase in p‐STAT3 nuclear translocation, while the combination of WP1066 and Alpinetin significantly blocked p‐STAT3 nuclear entry (Figure 3L). Together, these findings suggested that Alpinetin's antiinflammatory role were brought about via the targeting of the JAK2/STAT3 pathway.

### 3.3 Alpinetin inhibited activated BV2 cell‐mediated neuronal apoptosis

**FIGURE 4:**
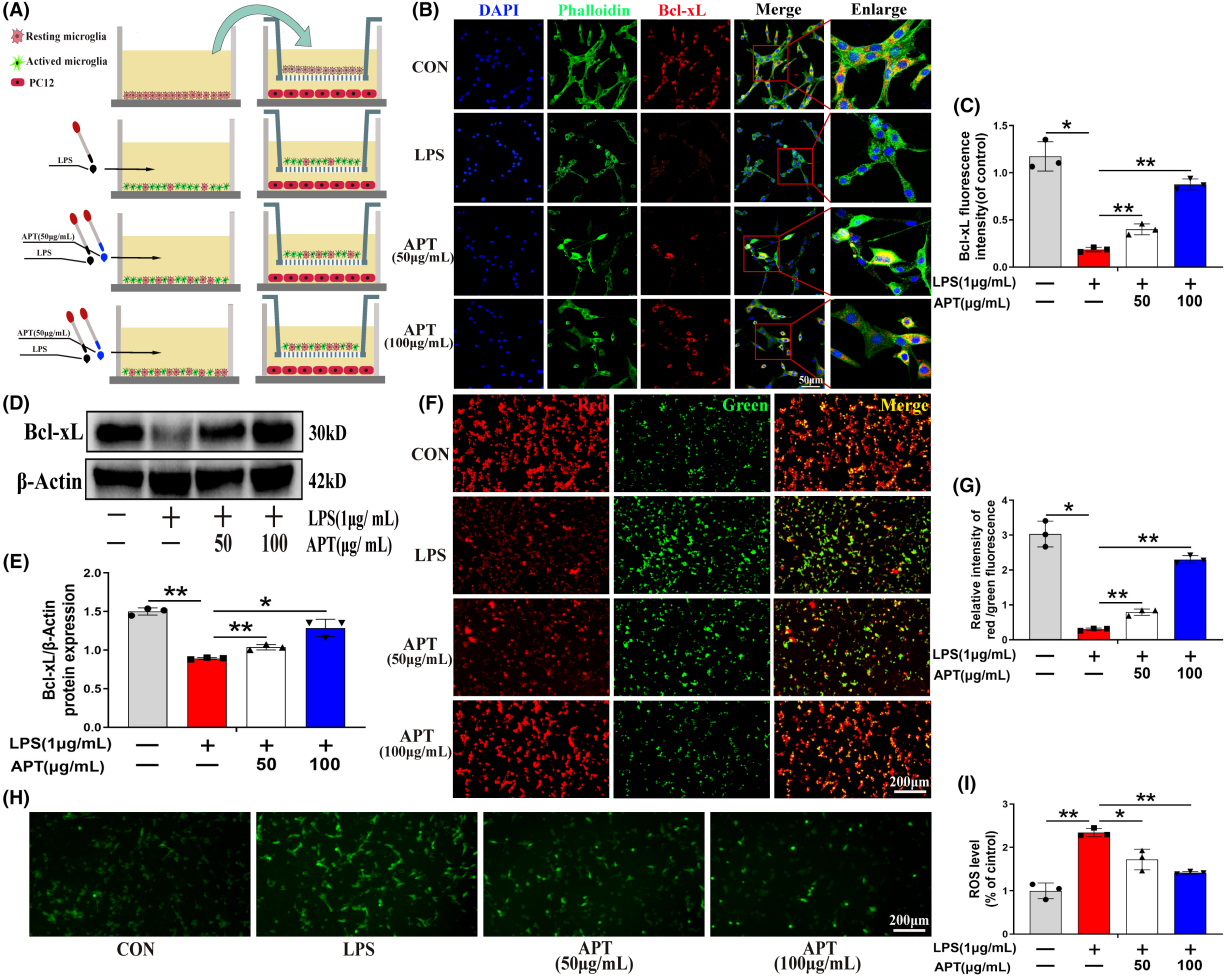
Neuroprotection of APT against activated microglia‐mediated neurotoxicity. (A) Schematic diagram of co‐culture of BV2 microglia and PC12 cells. (B, C) Immunofluorescence staining and quantitative data revealed the expression levels of Bcl‐xL (red) and phalloidin(green) in each group of PC12 cells. (D, E) Western blot images and quantification showing Bcl‐xL protein levels in PC12 cells. (F) Changes in MMPs in PC12 neurons were captured by fluorescence microscopy. (G) Quantitative data analyzed the red/green ratio of PC12 cells. (H) Intracellular ROS detection with dichloro‐dihydro‐fluorescein diacetate (DCFH‐DA) obtained by immunofluorescence staining. (I) Quantification of the proportion of ROS‐positive PC12 cells. *N* = 3 per group for Western blot assay, *N* = 3 per group for Immunofluorescence staining assay. *p < 0.05 and **p < 0.01.

To evaluate the effect of Alpinetin on neuroinflammation‐induced neuronal apoptosis in vitro, we used a Transwell co‐culture assay (BV2 microglia and neuronal PC12 cells) as shown in Figure 4A. Microscopic observation showed that PC12 cells co‐cultured with untreated microglia (CON group) had longer synapses, similar to those seen in neuronal axons. In contrast, PC12 cells co‐cultured with LPS‐activated microglia (LPS group) displayed significant morphological changes (nuclear concentration, axonal contraction); however, pretreatment with Alpinetin (50 and 100 μg/ml; Alpinetin group) before exposure to LPS partially suppressed these changes in PC12 cell morphology (Figure S2A). We further assessed the effect of Alpinetin on neuronal apoptosis using calcein AM/PI double staining, with the results showing that Alpinetin could reverse activated microglia‐mediated neuronal apoptosis (Figure S2B,C; *p* < 0.01). Mitochondrial homeostasis is required for the maintenance of cellular physiological functions and the disruption of this balance can lead to cell apoptosis.^44^, ^45^ To ascertain whether Alpinetin exerts its neuroprotective effects through the stabilization of mitochondrial function, we determined the amounts of the Bcl‐xL protein, a member of Bcl‐2 family and connected to mitochondrial antiapoptosis, in PC12 cells using immunofluorescence. We found that the fluorescence intensity of Bcl‐xL in the LPS group was considerably lower than that in the CON group; however, this effect was significantly reversed in the Alpinetin group (50 and 100 μg/ml) (Figure 4B,C; *p* < 0.05). A similar trend in Bcl‐xL protein levels was detected using Western blotting (Figure 4D,E; *p* < 0.01). We further measured the MMP in the three groups using a JC‐1 assay kit and found that Alpinetin treatment led to a concentration‐dependent recovery of the MMP (reflected by the relative red/green fluorescence intensity) in co‐cultured PC12 cells (Figure 4F,G; *p* < 0.05). It has been reported that abnormal ROS production induces neuronal apoptosis.^46^, ^47^ As shown in Figure 6H,I, neuronal ROS levels were increased in the LPS group relative to those in the CON group; however, ROS accumulation was significantly inhibited following Alpinetin pretreatment (reductions of 26.5% and 39.5%, respectively, for the Alpinetin concentrations of 50 and 100 μg/ml) (*p* < 0.05). Combined, these data confirmed that Alpinetin exerts significant neuroprotective effects by inhibiting activated microglia‐mediated neuronal apoptosis, mitochondrial dysfunction, and ROS production.

### 3.4 Alpinetin ameliorated rat pathology and motor function after SCI

To assess the biosafety of Alpinetin administration after SCI, after 56 days of surgery, we collected the main organs (heart, liver, spleen, lungs, and kidneys) for H&E staining. No significant differences in the morphology of the organs were observed between the Alpinetin and Sham groups (Figure S3), confirming that Alpinetin treatment was not toxic to rats.

**FIGURE 5:**
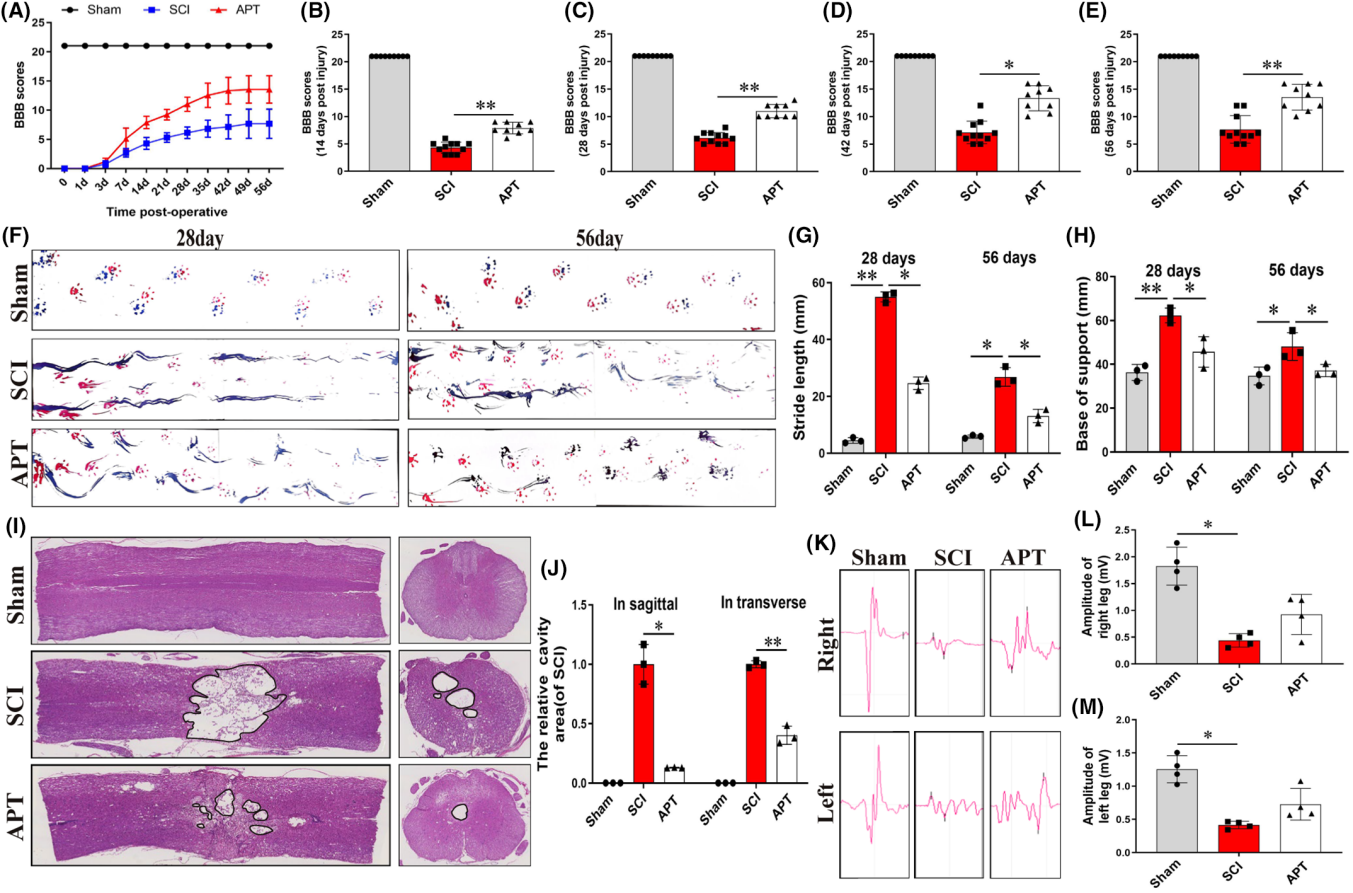
Alpinetin improves the recovery of motor function in rats after spinal cord injury. (A) Basso‐Beattie‐Bresnahan (BBB) scores of different groups of rats at different time points after injury. (B–E) Quantitative analysis of the BBB score at 14, 28, 42, and 56 days after injury. (F) Footprint analysis to assess hindlimb motor function recovery. Forelimb footprints are shown in red and hindlimb footprints are shown in blue. (G, H) Quantification of stride length and base of support to assess motor recovery at 28 and 56 days postinjury. (I, J) Image and quantification of H&E‐stained cavity areas in longitudinal and transverse sections at 56 days postinjury. (K) The electrophysiology of each group was detected. (L, M) Quantitative analysis of amplitudes of motor evoked potential in each group. *N* ≥ 9 per group for BBB score, *N* = 4 per group for footprint assay, *N* = 3 per group for H&E staining of longitudinal and transverse sections at 56 days postinjury. *p < 0.05 and **p < 0.01.

Next, we evaluated whether Alpinetin can promote motor function recovery in rats after SCI using the BBB locomotion rating scale, footprint analysis, and electrophysiology. All the rats exhibited normal hindlimb motor behavior (scores of 21) before injury. Immediately after spinal cord crush injury, however, the lower limbs of the animals developed complete paralysis (scores of 0) (Figure 5A). Within 7 days, rats in both the SCI and the Alpinetin groups showed only limited recovery. On Days 14 to 56, the BBB scores of rats that received Alpinetin treatment were significantly higher than those of animals in the SCI group (mean final BBB scores of 14 and 8, respectively) (Figure 5B–E; *p* < 0.05). These results showed that Alpinetin could significantly promote the recovery of motor function in rats following SCI. Figure 5F shows the results of the hind limb footprint analysis. During walking, both on Day 28 and Day 56, the footprints of the hind paws and the overlapping footprints of the front and hind paws of rats in the Alpinetin group were significantly clearer and more numerous than those in the SCI group, indicating that motor function and anterior and posterior limb coordination were significantly enhanced in the Alpinetin group relative to the SCI group. At both 28 and 56 days after SCI, rats in the Alpinetin group had significantly lower support bases and significantly shorter stride lengths relative to those of rats in the SCI group (Figure 5F–H; *p* < 0.05). This indicated that anterior and posterior limb coordination was significantly improved after Alpinetin treatment. Next, we used electrophysiological analysis to further evaluate functional recovery after SCI in rats of each group (Figure 5K). On day 56 after surgery, the signal amplitude of the right and left hindlimbs of rats in the SCI group was nearly 4‐ and 5‐fold lower than that of the Sham group, respectively (Figure 5L,M; *p* < 0.05). In contrast, the average signal amplitude recorded in the right and left lower extremities of rats in the Alpinetin group was 1.82 mV and 2.01 mV, respectively, significantly higher than that of rats in the SCI group (right: 0.44 mV, left: 0.42 mV). (Figure 5L,M; *p* < 0.05). These results showed that Alpinetin administration significantly improved the recovery of motor function in rats after SCI.

The histomorphology of the rat spinal cord after surgery is depicted in Figure 5I. On Day 56 post‐surgery, H&E staining of the longitudinal and traverse sections of the SCI group showed the presence of significant injury, structural disorder, and obvious cavities. In contrast, in the Alpinetin group, no significant area of injury was detected, and tissue cavity formation was distinctly decreased (Figure 5I, J; *p* < 0.05). These data suggested that Alpinetin markedly enhances spinal cord tissue regeneration and reduces the volume of cavities, leading to improved motor function recovery after SCI.

### 3.5 Alpinetin treatment after SCI inhibited microglial activation and reduced inflammation

**FIGURE 6:**
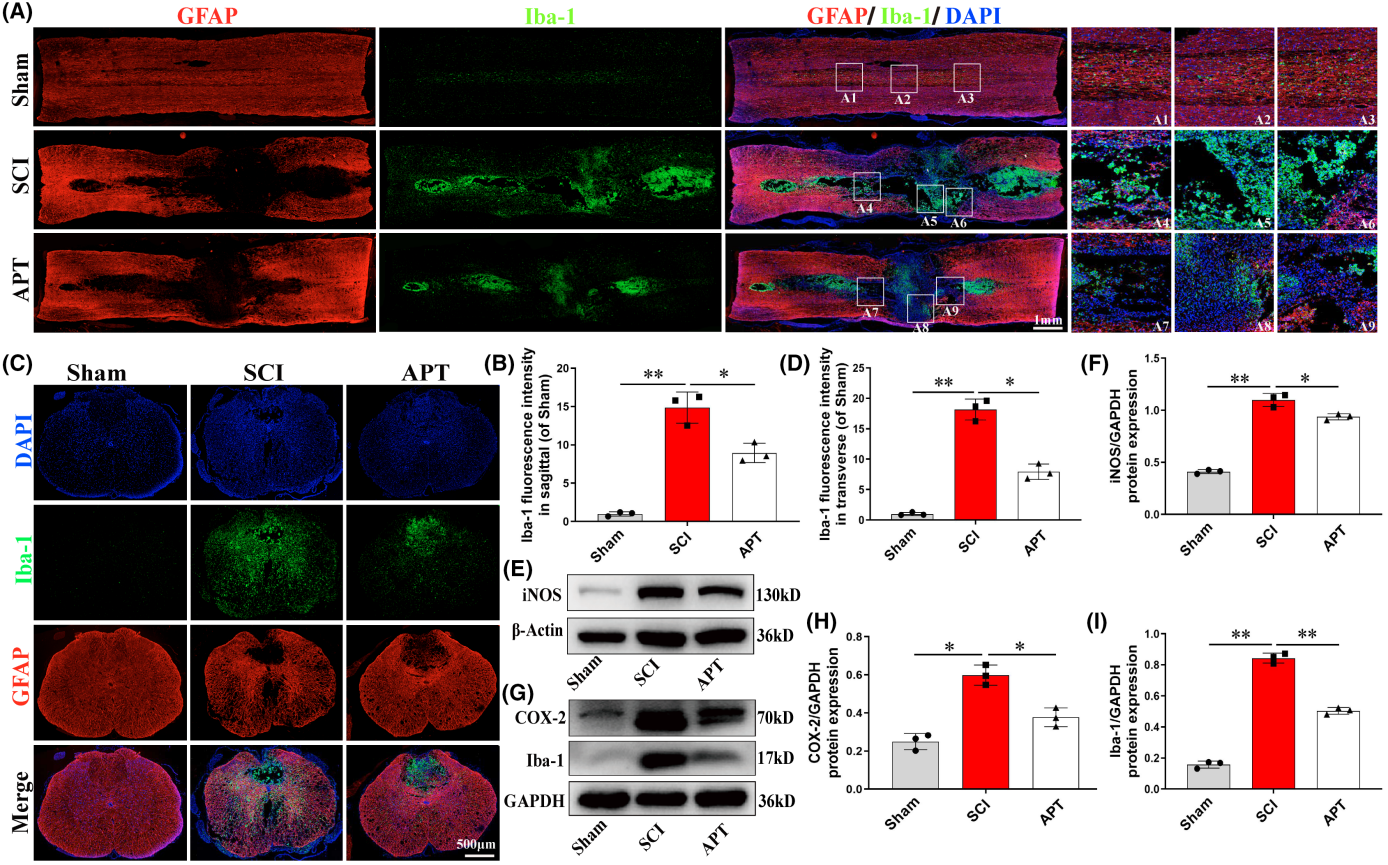
Alpinetin inhibits inflammatory response after spinal cord injury. (A, B) Image and quantification of Iba‐1 (green)/and GFAP (red) staining in longitudinal sections of spinal cords of rats in each group at 3 days postinjury. (C, D) Image and quantification of Iba‐1 (green)/and GFAP (red) staining in transverse sections of spinal cords of rats in each group at 3 days postinjury. (E–I) iNOS, COX‐2 and Iba‐1 proteins were detected and quantified in each group at 3 days postinjury. *N* = 3 per group for histology analysis, *N* = 3 per group for Western blot assay. *p < 0.05 and **p < 0.01.

GFAP is used to label astrocytes, which can be used to show the outline of the tissue after SCI.^3^, ^48^ Meanwhile, CD68 and Iba‐1 serve as surface markers for microglia and are commonly used to assess the degree of microglial activation. Here, to fully assess the effect of Alpinetin on microglial activation after SCI, we quantified the number of Iba‐1‐ and CD68‐positive (activated) microglia in longitudinal and transverse sections of spinal cord tissue by immunostaining. On Day 3 after surgery, the number of Iba‐1‐ and CD68‐positive microglia in and around the injury center was significantly lower in the Alpinetin group than in the SCI group (Figure 6A–D, Figure S4A–D; *p* < 0.05). Furthermore, compared with the Sham group, the expression levels of inflammatory mediators (iNOS and COX‐2) and those of activated microglia‐related proteins (Iba‐1 and CD68) in rats in the SCI group were significantly upregulated; however, these increases were significantly abrogated after treatment with Alpinetin (Figure 6E–I, Figure S4E,F; *p* < 0.05). Together, these results suggested that Alpinetin exerts a significant antiinflammatory effect by inhibiting the expression of inflammatory mediators as well as by reducing microglial overactivation. These results were consistent with the Alpinetin‐associated antiinflammatory effects observed in vitro (Figure 2).

### 3.6 Alpinetin mitigated neuronal death after SCI

**FIGURE 7:**
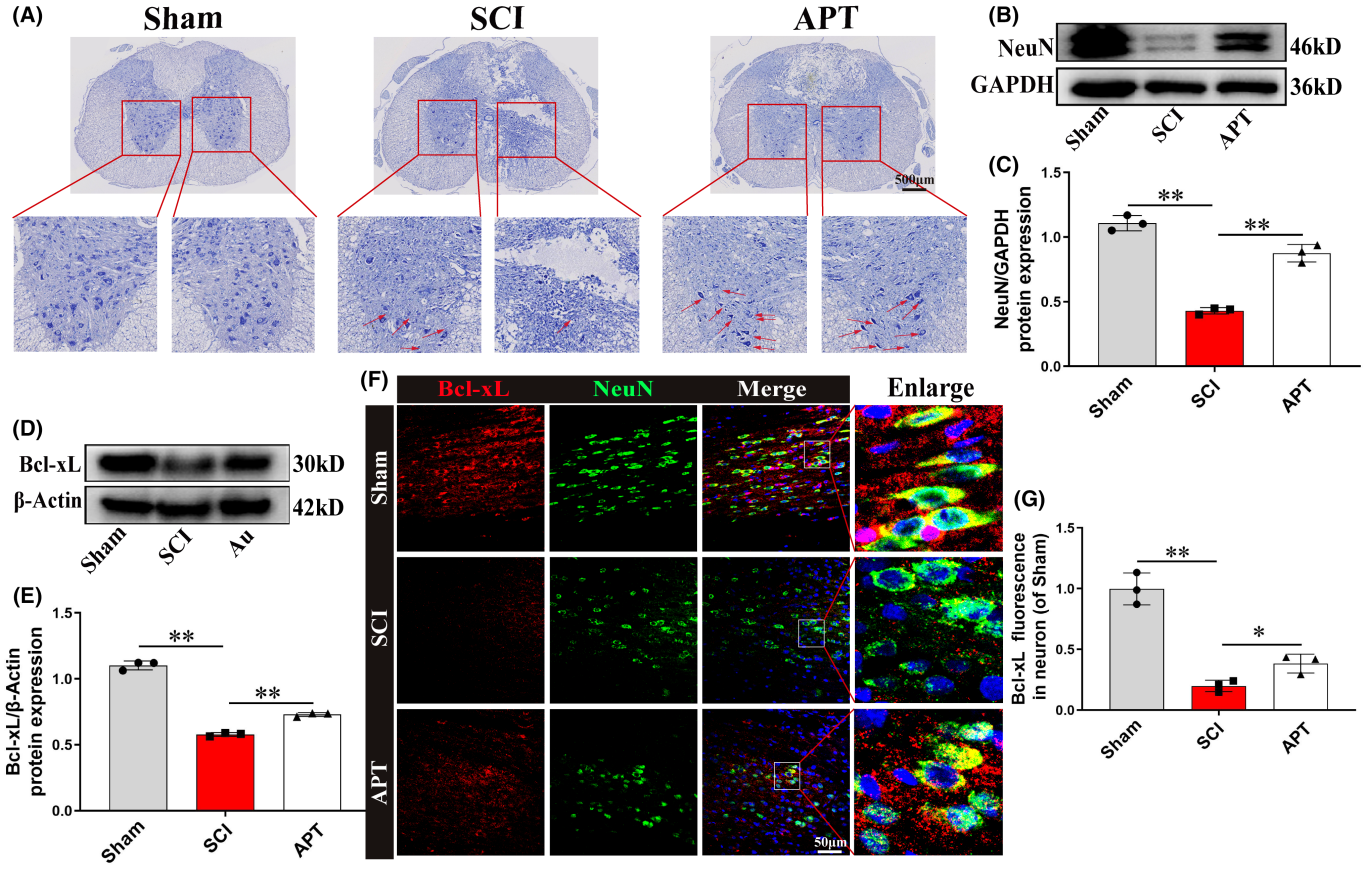
Alpinetin attenuates neuronal apoptosis after spinal cord injury. (A) Neuronal survival in transverse section on Day 3 postinjury was assessed by Nissl staining. (B, C) NeuN protein level were detected and quantified in each group at 3 days postinjury. (D, E) At 3 days after injury, the expression of antiapoptosis‐related protein Bcl‐xL was detected and quantified in each group. (F) Double immunofluorescence staining labeled NeuN (green) and Bcl‐xL (red) on longitudinally sectioned tissue in each group at 3 days postinjury. (G) Quantitative analysis of the fluorescence intensity of Bcl‐xL in neurons. *N* = 3 per group for histology analysis, *N* = 3 per group for Western blot assay. *p < 0.05 and **p < 0.01.

To confirm the neuroprotective effect of Alpinetin in vivo, we examined neuronal retention in spinal cord tissue through Nissl staining. On Day 3 post‐injury, there was a large loss of abdominal motor neurons (indicated by the red arrow) in the SCI group relative to that in the Sham group, an effect that was attenuated by Alpinetin treatment (Figure 7A). Moreover, Western blot analysis of spinal cord tissue showed that the levels of NeuN (a neuronal marker) were significantly higher in the Alpinetin group than in the SCI group (Figure 7B,C; *p* < 0.01), consistent with the Nissl staining and the in vitro neuronal apoptosis results (Figure 4). Next, we measured the level of apoptosis‐related proteins on Day 3 after SCI. Western blot (Figure 7D,E; *p* < 0.01) and immunofluorescence staining (Figure 7F,G; *p* < 0.05) analysis indicated that in comparison to SCI group, the Alpinetin group had a significantly greater protein expression level of the antiapoptosis‐related marker Bcl‐xL. In summary, our findings showed that Alpinetin can effectively suppress neuronal death in the damaged spinal cord.

### 3.7 Alpinetin improved axonal regeneration after SCI

**FIGURE 8:**
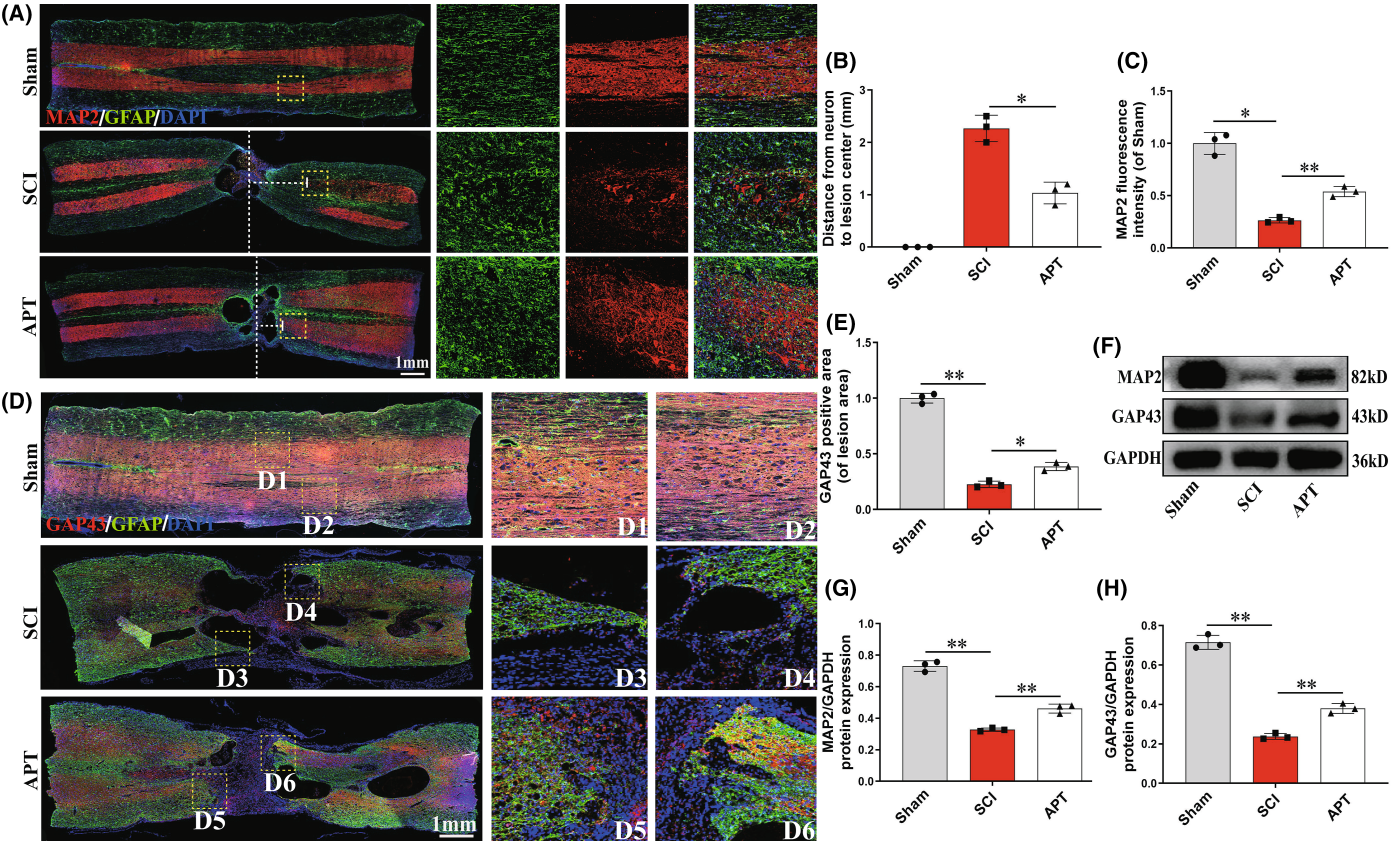
Alpinetin promotes axonal regeneration after spinal cord injury. (A) Double immunofluorescence staining showed GFAP (green) and MAP‐2 (red) in each group at 56 days postinjury. The vertical white dotted line represents the center of the lesion, and the horizontal dotted line length represents the distance from the injury center to the nearest neuron in the caudal. (B) Quantification of the distance from the neuron to the center of the lesion. (C) Quantifying the positive intensity of MAP‐2 from A. (D) Double immunofluorescence staining showed GFAP (green) and GAP43 (red) in each group at 56 days postinjury. (E) Quantification of GAP43 fluorescence intensity in the lesion area from D. (F–H) Western blot images and quantification showing MAP‐2 and GAP43 protein levels in the lesion area. *N* = 3 per group for histology analysis, *N* = 3 per group for Western blot assay. *p < 0.05 and **p < 0.01.

The restoration of motor function following SCI was assumed to be largely dependent on axonal regeneration.^49^, ^50^, ^51^ We used immunofluorescence to identify the expression of MAP‐2, an axonal microtubule component, in the damaged spinal cord in order to explore how Alpinetin affects axonal regeneration after SCI (Figure 8A). Compared with that in the SCI group, the distance from MAP‐2‐positive cells (neurons) to the center of the injury site (white dotted line) was reduced (Figure 8A,B; *p* < 0.05), while the number of MAP‐2‐positive cells was significantly increased (Figure 8A,C; *p* < 0.05). This indicated that Alpinetin can reduce neuronal loss and promote axonal regeneration in rats following SCI. To further evaluate the extent of axonal regeneration, nerve filament extension in the injured spinal cord was evaluated by GAP43 protein staining. We observed more neurofilaments in GAP43‐positive cells in the injury area of the Alpinetin treatment group than in that of the SCI group, and the neurofilaments broke through the glial scar to extend toward the center of the site of injury (Figure 8D,E; *p* < 0.05). We also detected MAP‐2 and GAP43 protein expression by Western blot and found that the expression levels of both proteins were obviously increased after Alpinetin therapy (Figure 8F–H; *p* < 0.01), in agreement with the results of immunofluorescence staining. Together, these findings confirmed that Alpinetin treatment helps to mitigate neuronal loss and stimulate axon repair after SCI, which improve the restoration of motor function after SCI.

## 4 DISCUSSION

Spinal cord injury is a serious, disabling disease that places a huge burden on patients and their families.^52^ Many studies have focused on mitigating secondary complications in SCI, such as neuroinflammation and neuronal apoptosis, to improve functional recovery.^53^, ^54^ Neuroinflammation is a response to CNS injury^55^ characterized by a strong inflammatory response mediated by activated microglia.^56^ Microglia act as the defense cells of the CNS, helping to maintain its homeostasis.^57^ However, microglia overactivation can result in the release of a variety of pro‐inflammatory mediators, which are thought to be markers and drivers of neuroinflammation‐related diseases.^58^, ^59^ Microglia‐mediated neuroinflammation is often associated with the abnormal secretion of ROS,^60^ which subsequently stimulate associated inflammatory pathways, thereby accelerating oxidative damage to cells and resulting in neuronal damage and death.^61^ In addition, neuronal impairment in neurodegenerative diseases can also lead to the activation of resting microglia,^62^ which exacerbates the neuroinflammatory response, induces MMP imbalance, and further enhances neuronal apoptosis.^63^ Accordingly, drugs that can inhibit microglial overactivation may also exert neuroprotective effects, causing increasing concern in microglia‐related neuroinflammatory diseases.

Studies have increasingly shown that some bioactive compounds extracted from Chinese herbal medicines can have a significant therapeutic effect on SCI.^6^ Alpinetin is a natural flavonoid with low systemic toxicity and a wide range of pharmacological activities, including antiinflammatory, hepatoprotective, cardiovascular protective, and neuroprotective effects.^30^ For instance, Wei et al. demonstrated that Alpinetin alleviated bone loss in mice by inactivating the PI3K and P38‐MAPK pathways, thereby reducing inflammation and ROS production.^25^ Additionally, Alpinetin was shown to have a marked antiinflammatory effect in LPS‐induced mastitis by blocking the TLR4/IκB‐α/NF‐κB pathway.^64^ Meanwhile, Wu et al. found that Alpinetin significantly reduced inflammation in a mouse model of allergic asthma via the modulation of the PI3K/AKT/NF‐κB and HO‐1 signaling pathways.^65^ However, the potential mechanisms underlying the antiinflammatory effects of Alpinetin in microglia and its neuroprotective effects in SCI are unclear. In our study, we showed that Alpinetin can bind JAK2 and inhibit its phosphorylation, and further demonstrated that Alpinetin can inhibit the release of pro‐inflammatory mediators via the inactivation of the JAK2/STAT3 pathway. Meanwhile, our results showed that Alpinetin treatment reduced abnormal ROS production and helped to normalize the MMP in PC12 cells co‐cultured with activated microglia, thus exerting a neuroprotective function. Furthermore, Alpinetin was found to significantly alleviate microglia‐mediated neuroinflammatory responses and neuronal apoptosis after SCI, thereby promoting nerve axon regeneration and enhancing motor function recovery in a rat model of SCI.

The JAK/STAT pathway consists of two protein families—JAKSs (JAK1, JAK2, JAK3, and TYK2) and STATs (STAT1, STAT2, STAT3, STAT4, STAT5a, STAT5b, and STAT6)—the members of which can be activated by a variety of inflammatory signals, including LPS.^66^, ^67^ Ligand binding to the cytokine receptor results in the transphosphorylation and activation of associated JAK proteins. The activated JAK/receptor complex provides a site for the docking and subsequent phosphorylation of STAT proteins by JAK. Phosphorylated STATs translocate to the nucleus, where they initiate a signaling cascade that exacerbates the neuroinflammatory process.^40^, ^66^ JAK2 and STAT3 are key members of the JAK/STAT pathway and play an important role in the pathophysiology of neurodegenerative diseases.^67^ For instance, it has been reported that JAK2 and STAT3 inhibitors can block an increase in pro‐inflammatory protein levels following ischemic brain injury.^66^ Additionally, the JAK2/STAT3 pathway is increasingly recognized as playing a key role in activated microglia‐mediated neuroinflammation as well as representing an effective therapeutic target in neuroinflammation.^68^ In this study, we identified the JAK2 protein as a target of Alpinetin. Molecular docking analysis showed that Alpinetin can bind well to the active pocket of JAK2 and effectively inhibit JAK2 and STAT3 phosphorylation in activated microglia without affecting total JAK2 and STAT3 protein levels. In addition, the inhibition of JAK2/STAT3 pathway activity by WP1066 significantly reduced the LPS‐induced increase in the phosphorylation levels of JAK2 and STAT3 and the protein levels of iNOS and COX‐2. Additionally, the combination of WP1066 and Alpinetin further inactivated the JAK2/STAT3 pathway and suppressed neuroinflammation. These findings suggest a novel mechanism in which Alpinetin targets JAK2 and inhibits its phosphorylation, thereby inactivating the JAK2/STAT3 pathway and, consequently, exerting antiinflammatory effects. However, further studies are needed to confirm the interaction between Alpinetin and JAK2, such as surface plasma resonance, biolayer interferometry, thermal shift assays, cell‐free enzyme activity experiments, microscale thermophoresis, and biolayer interferometry.

## 5 CONCLUSIONS

**FIGURE 9:**
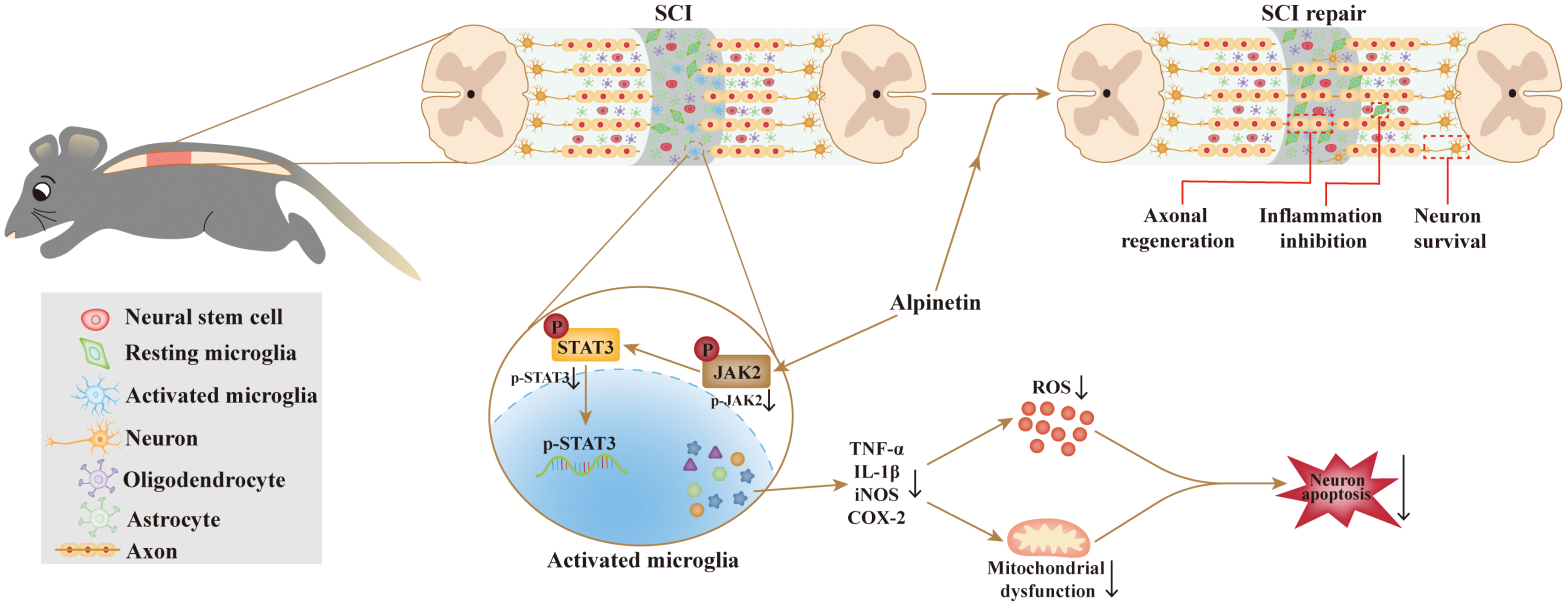
Alpinetin's therapeutic effects on spinal cord injury (SCI) are depicted in this diagram. Alpinetin alleviates the inflammatory response and neuronal toxicity caused by activated microglia via targeting the JAK2/STAT3 pathway, and ultimately promotes functional recovery in SCI rats.

In summary, we showed that Alpinetin inhibits microglial activation and the associated neuroinflammatory response by inhibiting the JAK2/STAT3 pathway. This alleviates neurotoxic factor‐mediated neuronal apoptosis, which ultimately enhances axon regeneration and promotes motor function recovery after SCI (Figure 9). These findings provide novel insights into the mechanisms underlying how Alpinetin inhibits SCI progression and suggest that Alpinetin may be a potential therapeutic agent for SCI.

## AUTHOR CONTRIBUTIONS

S. X. performed the whole study and wrote the manuscript. Y. Z. assisted with animal experiments and statistical analysis. Z. L. and A. L. helped in the methodology. W. T., X. X. and J. N. participated in animal experiments. N. Z. and G. Z. helped in Methodological analysis. J. L. and Z. L. oversaw the project and provided funding.

## FUNDING INFORMATION

This work was supported by Jiangxi Provincial Central Committee Guides Local Science and Technology Development Project (No. 20222ZDH04095), Jiangxi Province Traditional Chinese Medicine Science and Technology Program (No. 2020A0068), the “Double Thousand Plan” of Jiangxi Province, Natural Science Foundation of Jiangxi Province (Grant No. 20142BAB215046).

## CONFLICT OF INTEREST

The authors confirm that they have no conflict of interest.

## CONSENT FOR PUBLICATION

The authors have read the manuscript and agreed to publish.

## Supporting information


Figure S1.

Figure S2.

Figure S3.

Figure S4.

Figure Captions

## Data Availability

All data analyzed and presented in this study are available from the corresponding author on reasonable request.
