# Supplementary figures and images for: Alpinetin inhibits neuroinflammation and neuronal apoptosis via targeting the JAK2/STAT3 signaling pathway in spinal cord injury
Source: CNS Neurosci Ther. 2023 Jan 10;29(4):1094–108. doi: 10.1111/cns.14085 (PMC10018110; doi:10.1111/cns.14085)

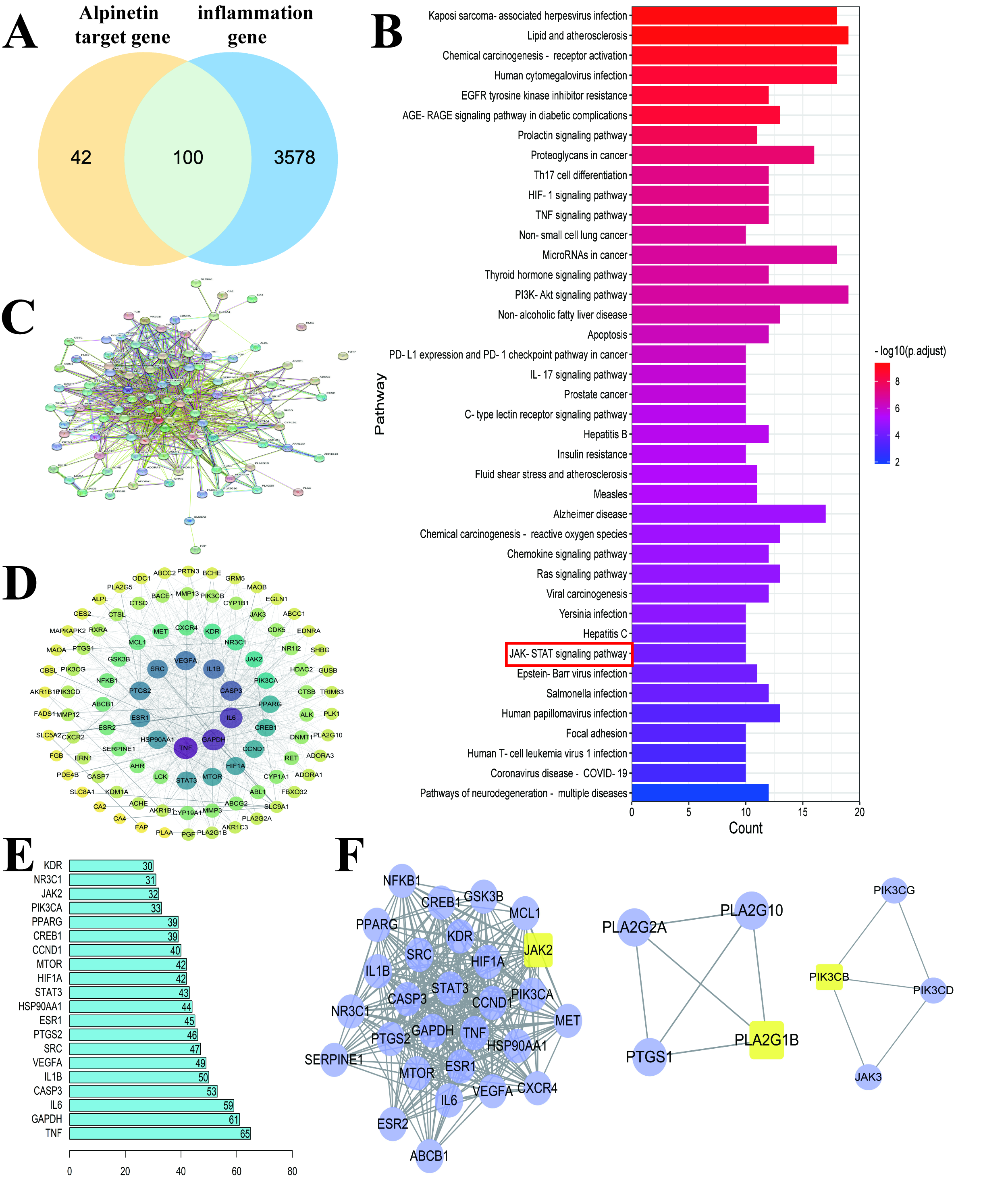

Supplement: Supplementary file 1 — Figure S1. [file CNS-29-1094-s003.tif]

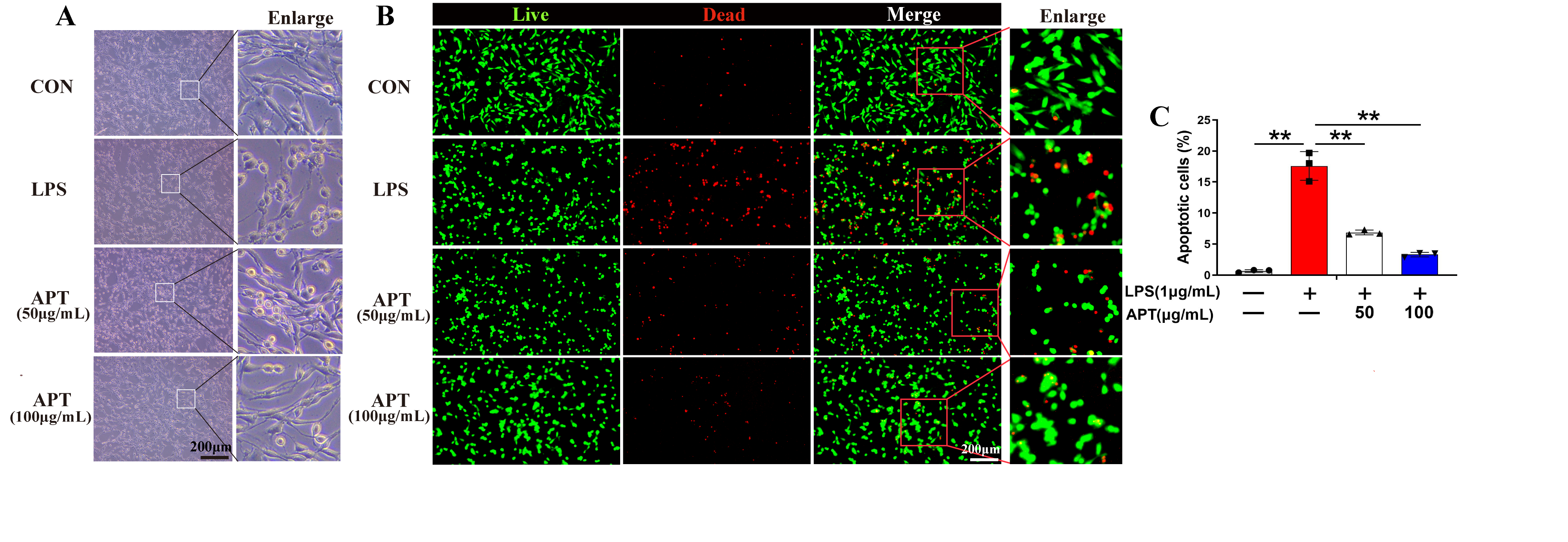

Supplement: Supplementary file 2 — Figure S2. [file CNS-29-1094-s002.tif]

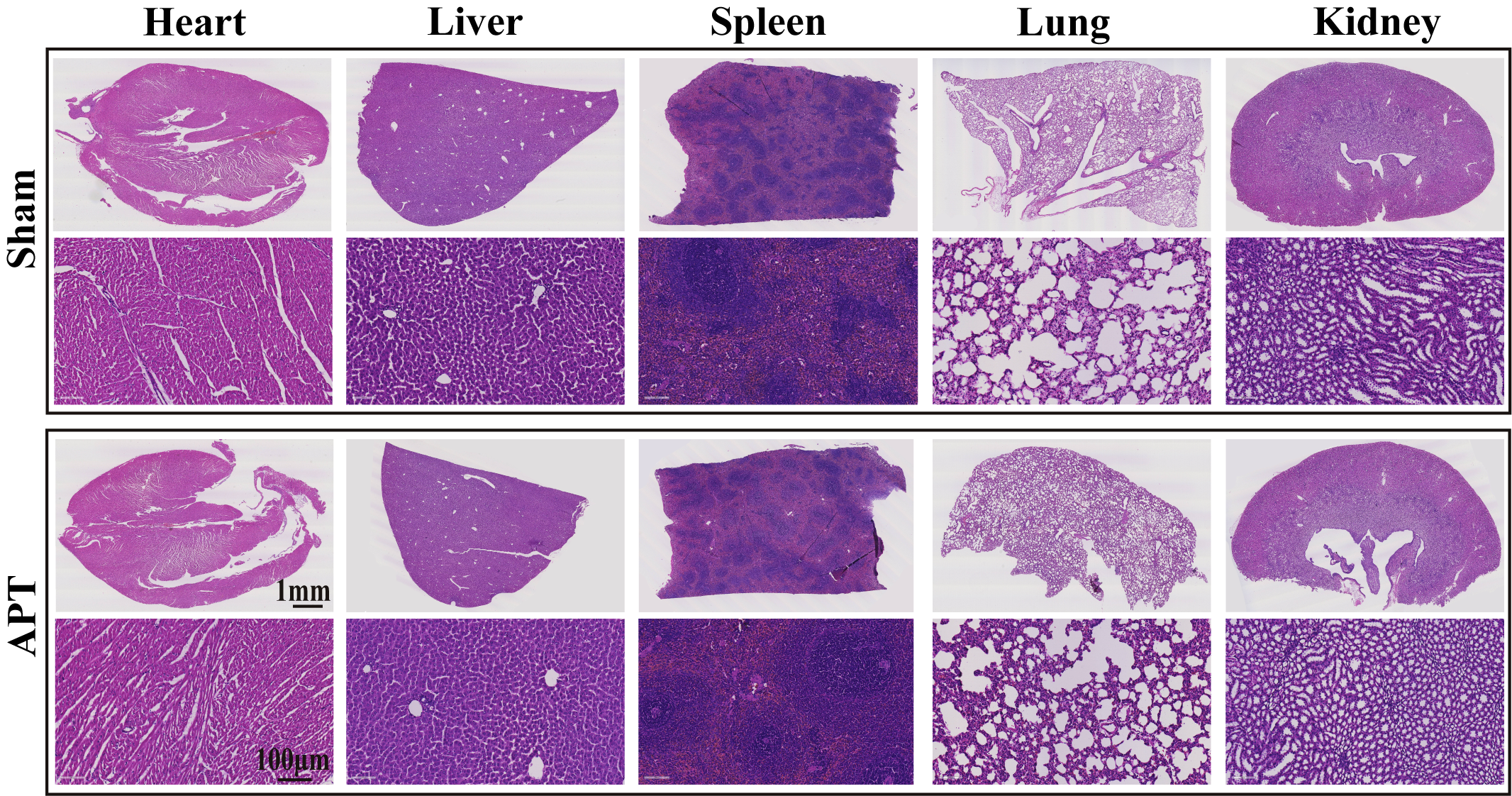

Supplement: Supplementary file 3 — Figure S3. [file CNS-29-1094-s001.tif]

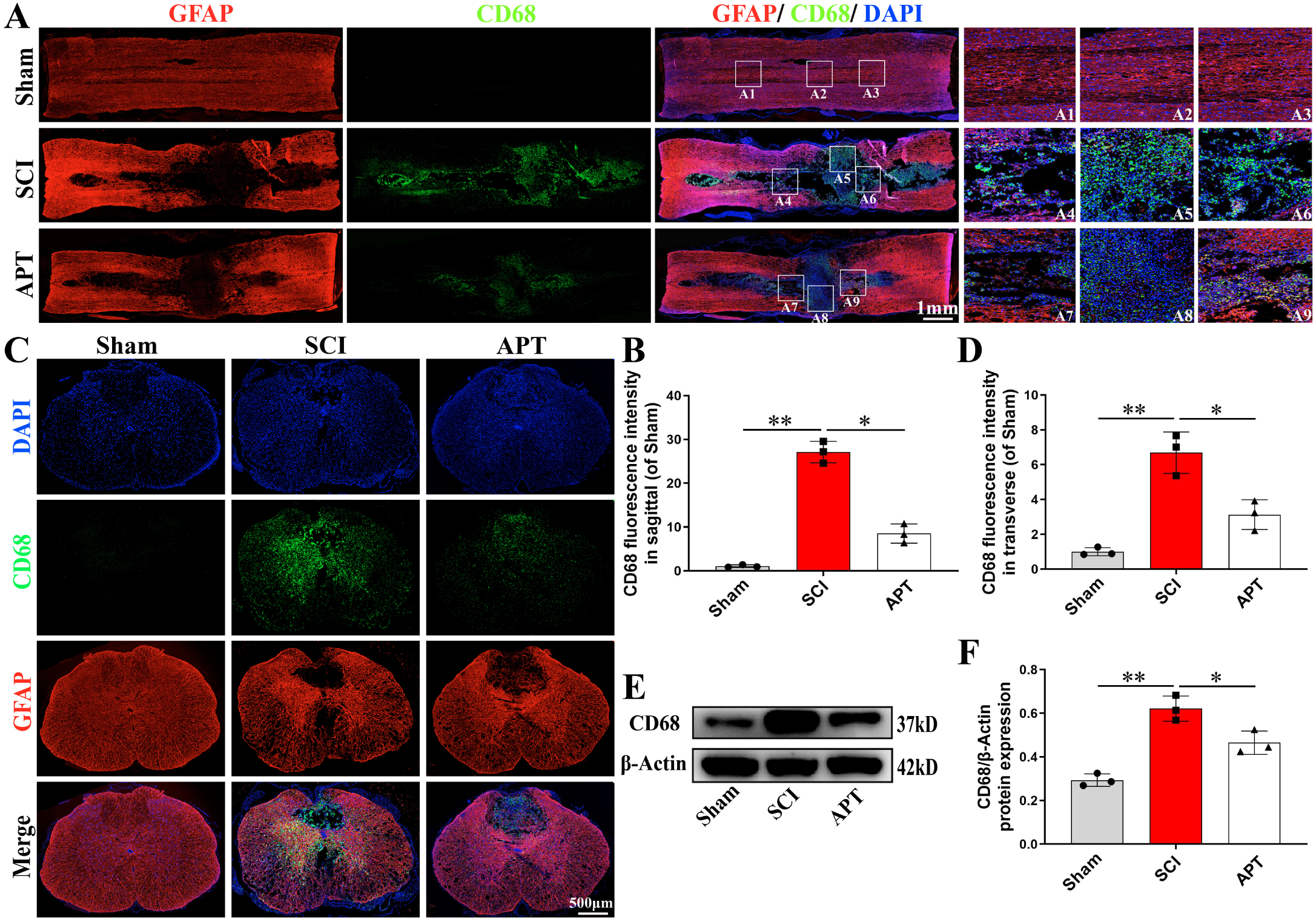

Supplement: Supplementary file 4 — Figure S4. [file CNS-29-1094-s005.tif]
