# Supplementary material for: Alpinetin inhibits neuroinflammation and neuronal apoptosis via targeting the JAK2/STAT3 signaling pathway in spinal cord injury
Source: CNS Neurosci Ther. 2023 Jan 10;29(4):1094–108. doi: 10.1111/cns.14085 (PMC10018110; doi:10.1111/cns.14085)
Supplement: Supplementary file 5 — Figure Captions [file CNS-29-1094-s004.docx]

**Fig** **S1**. Network pharmacology and bioinformatics analysis on Alpinetin. (A) The Alpinetin target proteins and inflammation-related proteins were intersected to find 100 common targets. (B) The KEGG pathway enrichment analysis was performed on the common targets of drugs and diseases. (C, D) Common targets of drug and disease were put into String website and Cytoscape software for PPI network analysis. (E, F) The PPI network was imported into Cytoscape software, and the key proteins were screened by topological analysis and MOCDE cluster analysis.

**Fig** **S2**. Alpinetin attenuates neuronal apoptosis induced by LPS-mediated activation of microglia. (A) Morphological changes of PC12 cells after co-culture of PC12 cells and microglia. (B) Dual labeling with Calcein AM/PI and quantification of live (green)/dead (red) in PC12 cells in each group. (C) Quantitative analysis of dead cells as a percentage of all PC12 cells. N = 3 per group for Immunofluorescence staining assay.

**Fig** **S3**. Biosafety Assessment of Alpinetin. Histopathology images of heart, liver, spleen, lung and kidney collected from 56 days postinjury. N = 4 per group for histology analysis,

**Fig** **S4**. Alpinetin suppresses inflammatory response after SCI. (A, B) Image and quantification of CD68 (green)/ and GFAP (red) staining in longitudinal sections of spinal cords of rats in each group at 3 days postinjury. (C, D) Image and quantification of CD68 (green)/ and GFAP (red) staining in transverse sections of spinal cords of rats at 3 days postinjury. (E, F) CD68 proteins were detected and quantified in each group at 3 days postinjury. N = 3 per group for histology analysis, N = 3 per group for western blot assay.
